# Being in a crowd bonds people via physiological synchrony

**DOI:** 10.1038/s41598-021-04548-2

**Published:** 2022-01-12

**Authors:** G. Baranowski-Pinto, V. L. S. Profeta, M. Newson, H. Whitehouse, D. Xygalatas

**Affiliations:** 1 Universidade do Estado de Minas Gerais, Belo Horizonte, Brazil; 2 Universidade Federal de Minas Gerais, Belo Horizonte, Brazil; 3 University of Kent, Kent, UK; 4 Oxford University, Oxford, UK; 5 University of Connecticut, Storrs, CT USA

**Keywords:** Human behaviour, Psychophysics

## Abstract

Collective events can generate intense emotions, shape group identities, and forge strong bonds. Do these effects extend to remote participation, and what are the psychological mechanisms underpinning their social power? We monitored psycho-physiological activity among groups of basketball fans who either attended games in-person (in a stadium) or watched games live on television in small groups. In-person attendance was associated with greater synchronicity in autonomic nervous system activation at the group level, which resulted in more transformative experiences and contributed to stronger identity fusion. Our findings suggest that the social effects of sports depend substantially on the inter-personal dynamics unfolding among fans, rather than being prompted simply by watching the game itself. Given the increasing prevalence of virtual experiences, this has potentially wide-reaching implications for many domains of collective human interaction.

## Introduction

Collective events at which emotions run high can have long-term psychological impacts, transforming individuals personally and forging strong group identities^1–3^, promoting pro-group behaviors^4^, and even leading to group violence and other extreme actions^5^. Recent technological and societal developments increasingly provide both opportunities and pressures for more remote event attendance^6,7^. As these current trends are here to stay^8^, it is important to ask the question: do remote events produce equivalently bonding experiences for attendants? To address this, we examined the psycho-physiological effects of attending sports matches among basketball fans, hypothesizing that in-person attendance would lead to a more intense, transformative experience for participants, because such experiences largely depend on the interpersonal dynamics among fans, rather than strictly on the properties of the game itself.

Although much research has focused on psycho-social aspects of collective arousal, little is known about its underlying physiology and how it relates to these inter-personal effects. Moreover, while there is evidence suggesting that shared arousal is a complex bio-social phenomenon^9^, integrated research examining physiological and social effects in tandem is rare. Studies have found that arousing social gatherings can lead to the synchronization of autonomic activity among participants, including spectators^10,11^, and that shared arousal is associated with intense pro-group sentiments^1,12–14^. However, as the literature concerning physiological synchrony is still under development, there is little theoretical precision or methodological consolidation^15^, and as a result the links between these analytic levels are not yet fully understood^16^.

In the present paper, we focus on the association between group physiological synchrony and relationship outcomes. There is growing evidence that personally transformative experiences, when shared with others, can lead to a particularly powerful form of social cohesion known as ‘identity fusion’^17^. Identity fusion is a form of group alignment in which personal and group identities are activated synergistically (activation of either identity makes the other more salient). In contrast, the social psychology literature also describes ‘group identification’, a process in which personal and group identities operate hydraulically (making one salient makes the other less accessible)^18–20^.

Fusion is thought to result from feelings of shared essence, stemming either from perceptions of shared biology or the sharing of personally defining experiences with other members of the group^5^. The latter ‘shared experiences’ pathway to fusion requires that a personally defining experience has a ‘transformative’ quality, in the sense of transforming one’s personal identity^1,5,17^. When transformative experiences that define the personal self are also shared with others, personal and group identities become fused together, creating the synergistic relationship between the two identities that is lacking in group identification^17^. A key question remains, however, as to what it means for a transformative event to be *shared*.

One option is that synchronized action constitutes a shared experience. Previous research has demonstrated that moving in synchrony can increase social attachment, positive feelings towards interaction partners, trust, and cooperation, and boost levels of endogenous opioids associated with social bonding^19,21–23^. In one study, for example, synchronized drumming was associated with the coordination of autonomic activity measured by inter-beat intervals in the drummers’ heart rates, as well as with increased feelings of cohesion among group members^24^.

Nonetheless, while shared action is a common way of bringing physiological states into alignment^25^, it is sensitive to socio-environmental input^26,27^, and is not in itself sufficient to produce this alignment. In fact, in some cases it may even have the opposite effect. For instance, a study of dyads engaged in a construction project using LEGO bricks found that movement synchrony was not associated with heart-rate synchrony, and that motor coupling was in fact associated with negative affective outcomes^28^. Additionally, the effects of shared arousal can over-ride or operate independently of motor synchrony. For example, a laboratory experiment found that skin conductance and electromyographic measures of synchrony were associated with group cohesion among participants who were engaged in different tasks^29^. Similarly, a field study investigating a fire-walking ritual found that heart rate synchronicity between pairs closely tracked social proximity, irrespective of participants’ role in the ritual^11^.

Paying attention to the socio-environmental context of these shared experiences may therefore be key to understanding how group cohesion comes about. For example, does sharing an event revolve around attending to the same stimuli and thus imagining that one’s emotions and thoughts are shared with others, or does it also involve more fundamental adjustments, such as the inter-personal synchronization of physiological states^30^? Further, how is this synchronization affected by sharing the experience directly, by being physically co-present, as opposed to indirectly or vicariously, by observing the same event remotely?

**Figure 1:**
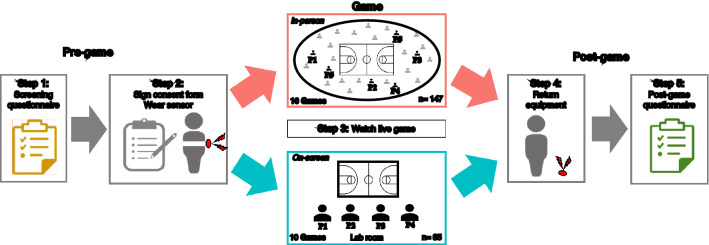
Summary of experimental procedures.

To address these questions, we analyzed data from 26 regular season games of NCAA Division I men’s and women’s basketball teams. We recruited 182 (86 female) fans of the UConn Huskies, a college team with one of the most loyal fan bases in the USA. Participants were assigned to one of two conditions, watching their team play live either in person at the home stadium, or remotely on a screen (Fig. 1). For the in-person condition, we recruited between four and fifteen participants per game, who attended a Huskies home game at the Harry A. Gampel Pavilion. Participants in this condition chose their own seats in the stadium and watched the games immersed in an audience of average size of 8344 (SD = 1520). For the remote condition, subjects watched a live televised transmission of a game on a large screen in groups of four on campus. None of the subjects in the remote condition knew each other before the experiment.

Using wearable heart rate monitors in both conditions, we recorded oscillations in autonomic nervous system activity as a physiological measure of arousal throughout the games. The devices also had in-built accelerometers, which we used to examine the effects of physical activity. Heart rate (HR) was chosen to measure synchrony due to both practical and theoretical reasons. Practically, HR provides a sufficient number of observations needed to perform our planned analyses^31^. Theoretically, HR relates to the function of both the sympathetic and the parasympathetic branches of the autonomic nervous system, making it ideal for lengthy and rapidly changing basketball games. Specifically, basketball is a long event (approximately 2 h) that involves frequent changes in score and numerous periods of inactivity in the form of timeouts, breaks, and clock stops. Fans therefore experience stressful moments with high fight or flight responses interspersed with periods of relative calmness. HR is a measurement that can capture the complexity of these fluctuations.

We used Multidimensional Recurrence Quantification Analysis (MdRQA) to quantify physiological synchrony among participants’ heart rates at each game. By physiological synchrony, we refer to the spontaneous temporal coordination or interdependency between the physiological activity of two or more individuals^15,31^. While previous studies on the synchronization of physiological signals have typically been limited to dyads^32,33^, MdRQA is a recurrence-based technique designed to measure the coordination of multiple variables across time by quantifying patterns of repetition in a multidimensional time-series dataset^34,35^. It captures non-linear relations, thus processing complex information that is not available in standard linear cross-correlation analyses.

**Figure 2:**
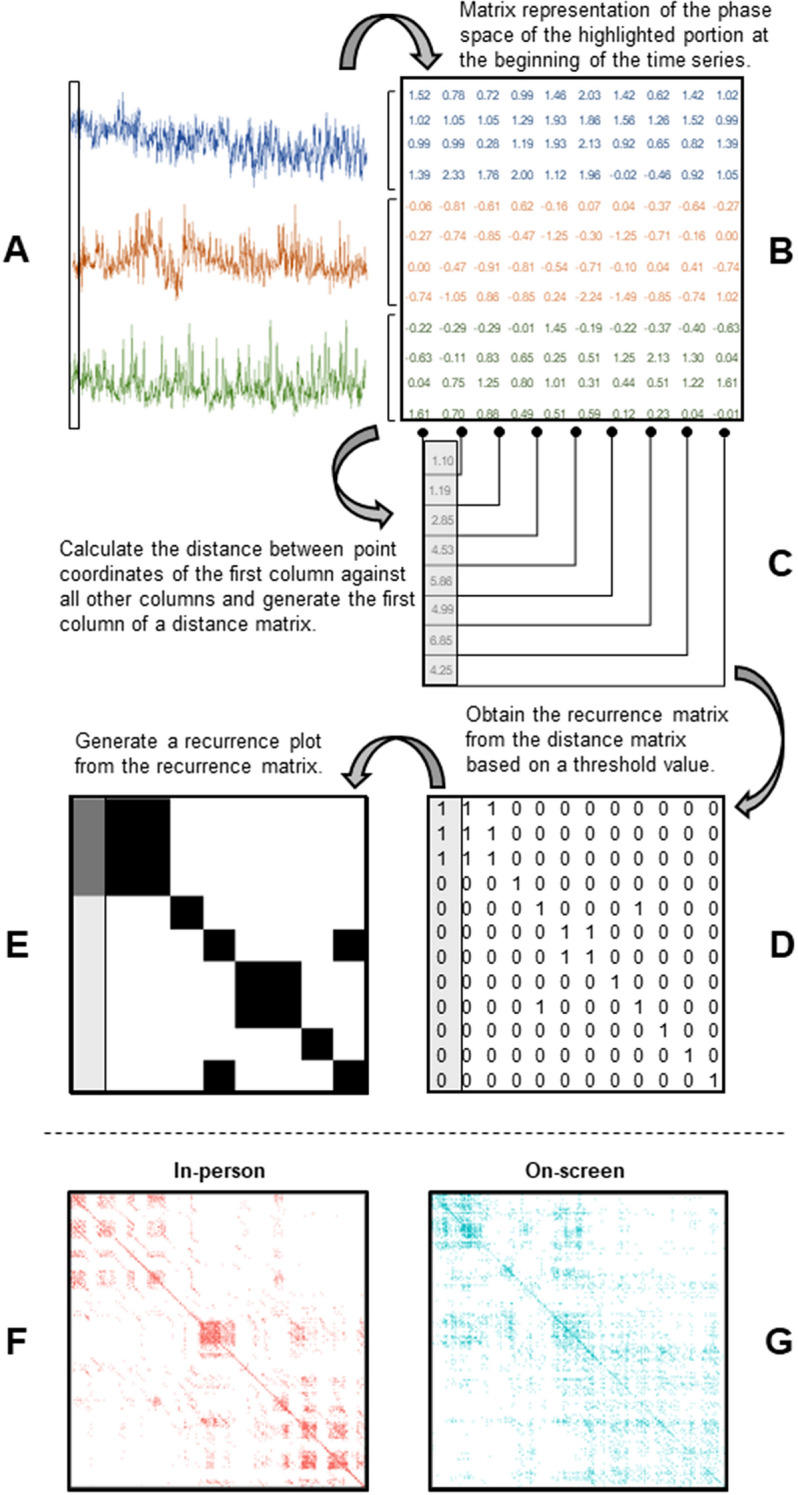
MdRQA procedures and outcomes. (**A**) Representative segments of three normalized heart rate time series. (**B**) State space matrix built from the combination of heart rate time series after the average delay and the average embedded dimension were obtained. Only the highlighted portion of the time series is shown in the phase space matrix. (**C**) Distances between the point expressed in the coordinates of the first column and the points with coordinates defined in the other columns of the phase state matrix. (**D**) Recurrence matrix obtained after applying a distance criterion to the distance matrix. When a cell in the distance matrix contains a value within this criterion, a “1” is assigned to its correspondent position in the recurrence matrix; otherwise, a “0” is assigned. (**E**) Recurrence plot obtained from the recurrence matrix. Where the recurrence matrix contains a 1, a black square is drawn. In turn, white squares are drawn for 0 s. The first column is highlighted to show its correspondence with the first columns of the recurrence and the distance matrices. (**F, G**) Representative recurrence plots for each condition.

MdRQA produces recurrence plots that allow the extraction of various recurrence measures that can be used to quantify group synchrony by examining the group as a dynamical system (Fig. 2). Although these measures are inter-related, each captures a different aspect of the dynamic relationship between the system’s variables. Here, we looked at the recurrence rate (REC), determinism (DET), and average diagonal line length (ADL) of the HR signals. REC assesses the repetitiveness of individual datapoints in the system’s phase space by calculating the percentage of points recurring within a certain radius. DET detects the percentage of recurrent points that are diagonally adjacent, providing an estimate of the system’s predictability by assessing whether its future states can be derived by its prior states. ADL, an estimate of prediction time, computes the average length of diagonal lines formed by recurrent points in the recurrence plot. A longer ADL indicates more persistent recurrence patterns within the system^35–37^. DET and ADL are derived from REC, which was fixed to be similar between conditions (see SM for details).

We also used a self-transformativeness scale^1,38^, which assesses the impact of collective events in shaping one’s core sense of self (Table S1). Finally, we used an identity fusion scale, which measures feelings of oneness with the group^18–20^. Based on existing models of identity fusion^5^, we predicted that attending games in the stadium would lead to greater physiological synchrony among fans than watching remotely, and that this synchrony would produce a more transformative experience, which in turn would mediate the relationship between synchrony and identity fusion following the game.

## Results

**Figure 3:**
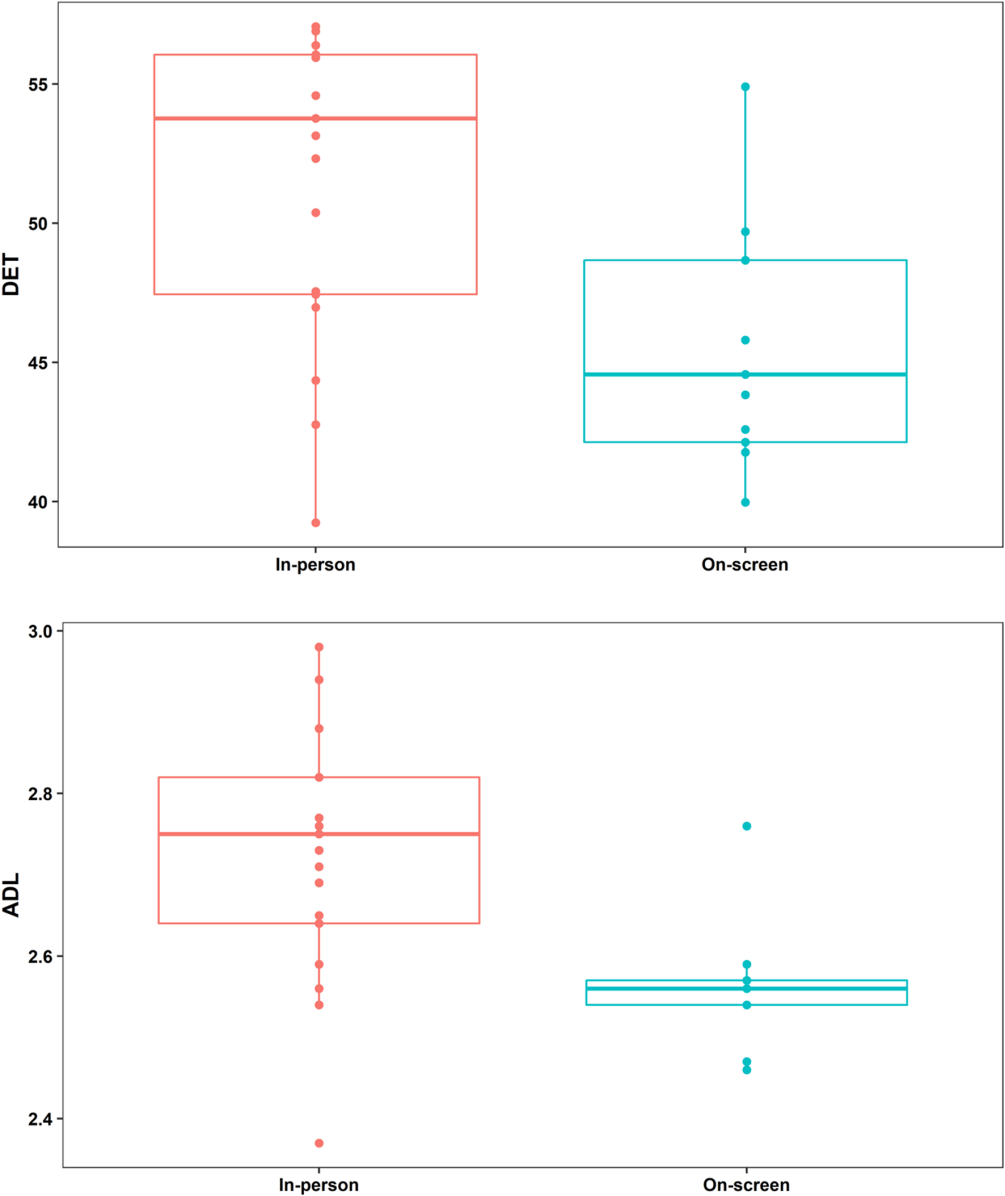
Boxplots of MdRQA outcomes. Large groups in the stadium displayed more physiological synchrony compared to small groups watching the games remotely.

**Figure 4:**
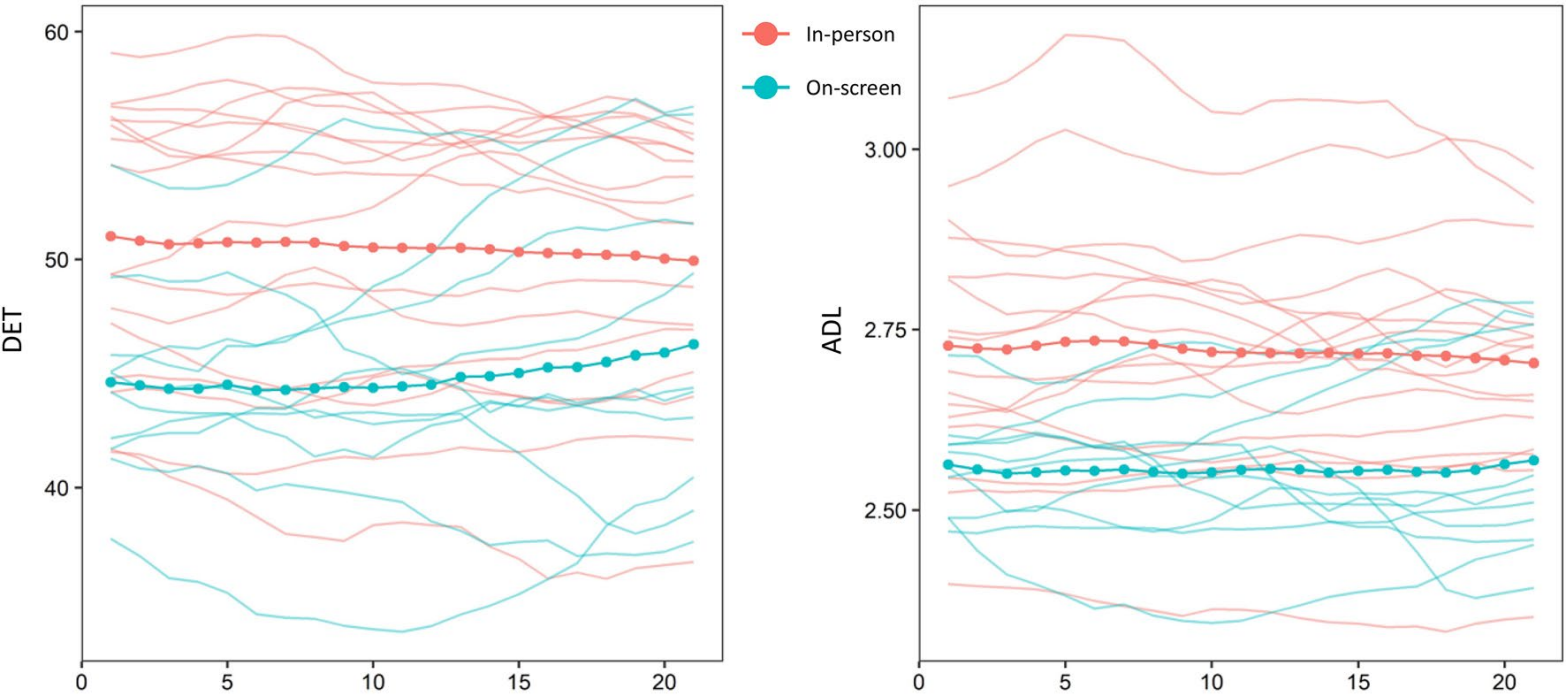
Windowed MdRQA. Each time series of each game was divided into 21 overlapping windows (horizontal axis) extending 80% of the length of the original time series.

We found that, compared to watching the game remotely, groups who attended the game in-person as part of a crowd displayed heart rate dynamics characterized by greater synchrony, as evidenced both by DET [*t*(22.235) = 2.768, *p* = 0.011, *d* = 1.089] and ADL [*t*(23.498) = 3.213, *p* = 0.004, *d* = 1.207]. This suggests that in-person attendance resulted in a stronger and more persistent alignment of heart-rate patterns. Rather than being confined to specific parts of the game, group synchrony remained higher in the in-person condition throughout the games, including the half-time interval (Figs. 3, 4). Crucially, these differences were not due to people being more physically active in the stadium: a multiple regression model with Condition and Activity (as assessed by the accelerometer measures) as predictors showed that watching a game on-screen was associated with lower ADL (*b*_1_ = − 0.218, *p* < 0.001), while activity was not (*b*_2_ = 0.152, *p* = 0.777). Similarly, watching a game remotely was associated with lower DET (*b*_1_ = − 8.865, *p* < 0.001), while activity was not (*b*_2_ = 9.518, *p* = 0.663).

Transformativeness correlated with both DET (r = 0.213, *p* = 0.004) and ADL (r = 0.217, *p* = 0.003). Analyses of covariance (ANCOVA) showed an interaction between condition and synchrony in predicting transformativeness, such that both DET [F(1, 178) = 6.59, *p* = 0.011] and ADL [F(1, 178) = 5.57, *p* = 0.019] were related to a more transformative experience, but only in the stadium (see SM). Moreover, transformativeness correlated with fusion after the game (r = 0.239, *p* = 0.001), which remained after adjusting for pre-game fusion levels (r = 0.183, *p* = 0.014). Using simple recurrence (RQA) metrics extracted from individual HR data, we found no relationship between transformativeness and intra-individual patterns of arousal. Moreover, while arousal levels (mean HR) were higher in the stadium (t(180) = 5.008, *p* < 0.001), arousal did not correlate with transformativeness—it was specifically *shared* arousal that had the self-transformative effects.

**Figure 5:**
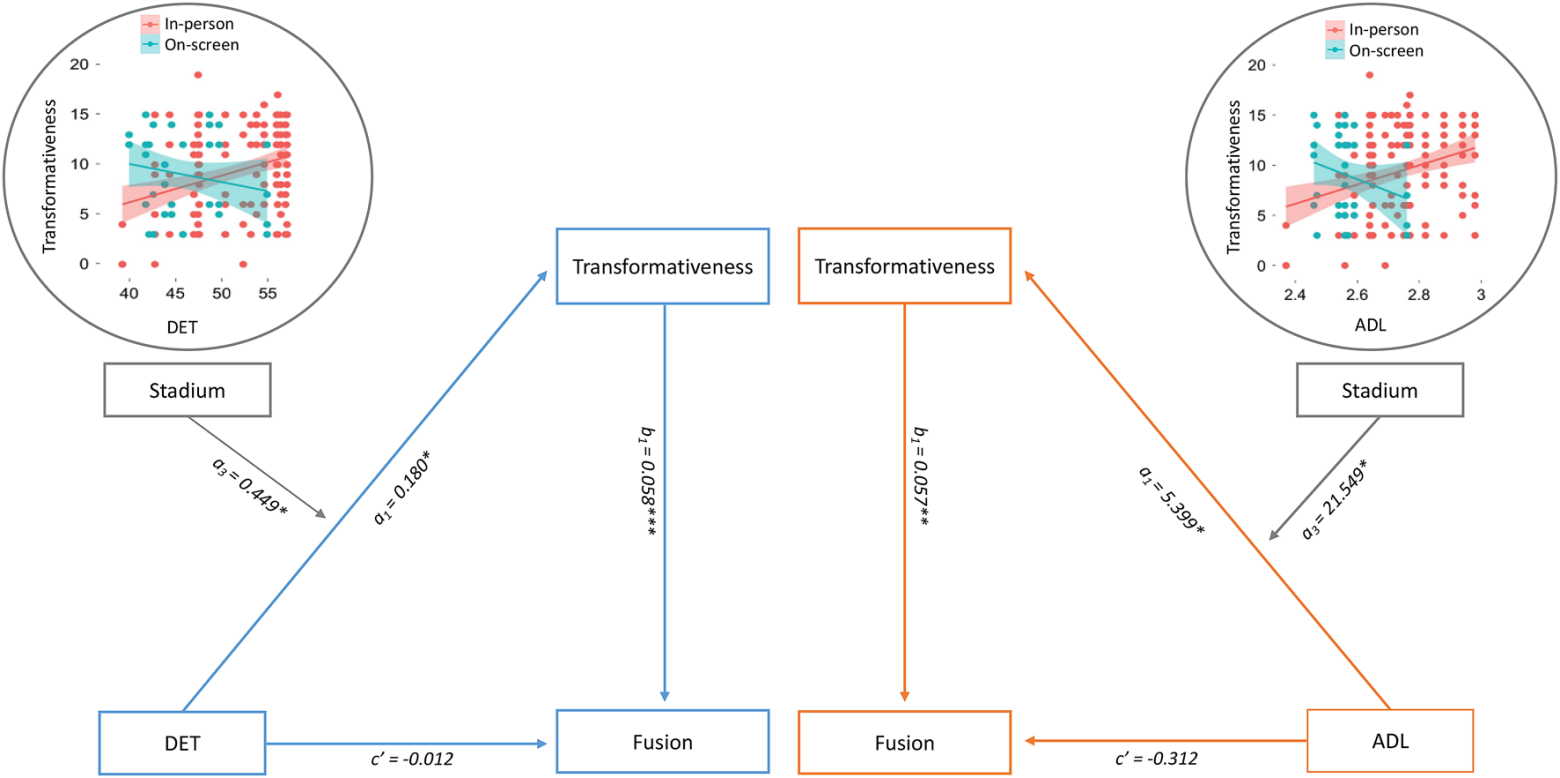
Moderated Mediation Model. Watching events in-person moderates the effect of group heart synchrony on self-transformativeness, which in turn leads to social bonding.

We found evidence of moderated mediation (Fig. 5), such that greater group synchrony (DET) led to higher self-transformativeness when spectators watched the game in-person at the stadium (b = 0.015, CI[0.005, 0.029]), as opposed to remotely (b = − 0.011 CI[− 0.031, 0.007]), and this synchrony in turn led to increased identity fusion (index of moderated mediation = 0.026, CI[0.005, 0.053]). This was robust controlling for a baseline measure of fusion before the game, as well as demographic variables and structural properties of the games. We re-ran the analyses replacing DET for ADL and the results for stadium (effect = 0.545, CI[0.165, 1.010.029]) and screen (effect = − 0.682, CI[− 1.933, 0.323]) conditions were slightly stronger (index of moderated mediation = 1.227 CI[0.073, 2.761]), (see Supplementary information).

## Discussion

Our findings suggest that the experience of attending collective events in-person, as part of a large crowd, is quite different from watching events remotely in small groups. These differences are measurable at physiological, psychological, and social levels. Compared to viewing remotely in small groups, stadium attendance in our study was associated with more transformative experiences, which mediated the relationship between heart rate synchrony and social bonding. Specifically, we found that in-person attendance resulted in greater physiological synchrony at the group level, and this synchrony predicted the upstream psychological impact of the game on feelings of transformativeness and group bonding. Rather than being due to individual arousal states or a mere result of the structure of the events themselves, these participatory effects are primarily social, shaped and constrained by the affordances of the socioecological context. The ability of sports and other collective events to inspire and mobilize crowds seems to rely upon the emergent inter-personal dynamics that unfold among fans present in the stadium, an effect which is experienced differently by those who witness the same event remotely.

Further insights into these differences of experience are provided by the fact that HR synchrony was related to transformativeness only in the stadium, suggesting that even when people did exhibit physiological synchrony in the remote condition, they did so in less personally meaningful and memorable ways. This is also in accordance with findings from studies showing that directly shared experiences correlated with stronger fusion than vicariously shared ones^39^. Future research should attempt to clarify more precisely why this is the case. For example, are spectators watching a game in the stadium exhibiting more closely shared emotional reactions during peak moments of the game, while those sitting in the same room are more likely to synchronize during commercial breaks or conversations? While MdRQA cannot capture such fine-grained oscillations in synchrony, we hope that new analytical techniques will make it possible to explore this in the future.

Our study compared two of the most common ways of viewing sporting events: (a) being immersed in a large crowd in the stadium and (b) watching remotely with a small group of people. Our design allowed us to collect the stadium data in a real-life setting. However, for the small-group setting, we chose a more controlled environment on campus to avoid confounds related to prior relationships between viewers. Future studies should investigate how watching a game at home with one’s friends and family compares to watching a game in a stadium. We predict that we would find similar effects, with stadium attendance resulting in greater physiological synchrony and more transformative experiences.

Meanwhile, there are also various intermediate conditions, which are beyond the scope of our study, but may be explored in future work. For example, how does watching a game in a sports bar or being in a fan zone compare to watching it in a stadium? We expect that effects will scale up as crowd size increases, although this relationship may not be linear, plateauing after a certain group size threshold is reached. Gatherings among fans outside of traditional sports settings may also prove informative for our understanding of group arousal, synchrony, and social bonding. Those may occur, for example, in the context of protests or pre-game rituals. We expect that such gatherings will also produce transformative experiences, although the lack of a common external stimulus (i.e. the game) may result in reduced physiological synchrony.

Our sample consisted of fans who had previously experienced live events and were largely already fused with the group. Anthropological theories^3^ and field studies^11^ suggest that prior group affiliation increases susceptibility to the effects of shared arousal. To better understand the formation of group cohesion, further research may specifically focus on first-time participants. Moreover, future studies may delve deeper into specific properties of in-person attendance that underlie these effects. For instance, are these results affected by people’s sense of agency (whether actual or perceived) in potentially impacting the event? Other factors of interest might include sensory arousal (e.g. noise levels), shared movement patterns, and the display of symbolic markers that could strengthen group identities. All of these factors may interact to produce an enhanced experience for participants, and more targeted studies are needed to disentangle the paths to social bonding.

The findings reported here have implications for all kinds of collective events beyond the domain of sports, including theatrical performances, musical concerts and festivals, academic conferences, and political rallies and demonstrations. Such events vary in the degree of active participation involved. In particular, events such as religious rituals, dance parties, choral singing, and other taxing group activities often involve stronger activation of personal agency than that required of mere observers of an event. The impact of variation in participatory aspects on transformativeness and fusion are obvious topics for future investigation. As these types of events increasingly occur remotely, a better understanding of their psycho-social effects and their underlying mechanisms may help harness their full potential to bring people together and create meaningful experiences.

## Methods

### Participants

We recruited fans of UConn’s NCCA Division I men’s and women’s basketball teams. Prospective participants were contacted and invited to engage in the study via various methods of recruitment, including advertisements posted in public places inside and outside the university campus, fan-related social media accounts, and nominations of fans by other fans in a snowball approach. The study was approved by the Institutional Review Board of the University of Connecticut (protocol # H16-212), and all methods were performed in accordance with relevant guidelines and regulations. All study participants provided informed consent.

### Conditions

Participants were randomly invited to watch live regular season games of UConn’s NCCA Division I men’s or women’s basketball team, either in person or remotely. In the In-person condition, subjects attended a home game at the Harry A. Gampbel Pavilion, home of the UConn Huskies. In the Remote condition, subjects watched a live transmission of one of the team’s away games on a large (102″) screen on campus. Participants in the Remote condition watched the game in groups of four. In the In-person condition, we recruited between four and fifteen participants per game. Participants in that condition were seated randomly across the stadium, based on the seating area in their tickets. The study ran over a total of 26 games (16 In-person and 10 Remote).

### Materials and procedures

Before the game, participants completed a Screening Recruitment Questionnaire (SRQ) used to ensure eligibility for the study. In addition to demographic information, the SRQ included four adapted scales: a *Basketball IQ Test*^40^, a 4-item instrument aimed to assess participants’ understanding of the game; a 3-item *Sport Spectator Identification Scale* (SSIS)^41^ aimed to assess participants’ level of fanship; a 7-item *Identity Fusion Verbal Scale*^42^ aimed to assess baseline identification with the team; and four questions about the *Frequency of Game Attendance*, where participants reported the number of men’s and women’s games they attended in the current and previous year.

On each game day, enrolled participants met with the experimenters in an on-campus room in the vicinity of the stadium within one hour before the game started. Upon entering the room, they read and signed a consent form and wore a heart rate monitor. In-person groups then walked to the stadium, located across the street. After the end of the game, they returned to this room, where the equipment was removed and a post-game survey was administered. Those in the remote condition watched live televised games in a room fitted with a 102-inch screen and sound system. To ensure that there were no prior relationships between these participants, we enrolled six subjects per game. Upon arrival, we asked subjects in each group whether they happened to know one another. If they did, we re-assigned some of them to a different session, so that there were no linked pairs within groups. Whenever no acquaintance was reported, the last two persons to arrive were reassigned to the next group.

All participants completed the post-game survey which included a *Transformativeness Scale*^1^*,* a 3-item instrument intended to measure the extent to which an event has shaped the participant as a person; and a *Pictorial Identity Fusion Scale*^18^. The pictorial scale measures the same construct as the verbal fusion scale used as a pre-game baseline (*r* = 0.52, p < 0.001), but we used different administration methods to avoid anchoring effects.

### Physiological measurements

We used the Zephyr BioHarness3 Model BH3, a wearable ECG sensor, to measure autonomic arousal levels during the game. This is a lightweight, unobtrusive device fitted around the trunk with a chest strap and worn inconspicuously under the clothes. Heart rate data extracted from the ECG were collected at 250 Hz rate. The sensor also records data from a 3-axis accelerometer, sampled at 100 Hz and band pass filtered to remove non-human artefacts and gravity. The root mean square of the three axes provides a measure of activity. Participants wore the monitors throughout the entire game.

### Data processing and analysis

Mediation analyses were conducted using Model 7 in Hayes’ PROCESS macro for SPSS v27^43^. All other analyses were conducted in RStudio^44^ using the following packages: chron_2.3–56, crqa_2.0, dplyr_1.0.2, ggplot2_3.3.2, ggpubr_0.4.0, lubridate_1.7.9, magrittr_1.5, Matrix_1.2–18, nonlinearTseries_0.2.10, plyr_1.8.6, rstatix_0.6.0, and tseriesChaos_0.1–13.1.

### Demographics

After excluding 73 participants with missing HR data, (Missing data may occur for different reasons, e.g. because the chest strap was not tight enough, a participant removed the device, the hardware malfunctioned, or the participant was too skinny, which may sometimes create problems with the fit of the strap.) we were able to analyze data from 182 subjects (147 in the In-person condition and 35 in the Remote condition). Participants in the two conditions did not differ significantly in terms of sex (χ^2^ (1,182) = 0.041, p = 0.839) or education (*t*(61.17) = 0.846, *p* = 0.401, *d* = 0.15), although those in the Remote condition were on average younger (*t*(175.28) = 3.419, *p* < 0.001, *d* = 0.42). There were no differences between conditions in terms of basketball knowledge (*t*(68.15) = -1.420, *p* = 0.160, *d* = -0.24), fanship level (SSIS) (*t*(53.99) = 0.851, *p* = 0.399, *d* = 0.16), game attendance (*t*(47.48) = 0.188, *p* = 0.852, *d* = 0.04), or baseline identity fusion (*t*(58.44) = 0.197, *p* = 0.845, *d* = 0.04). See Table S1 for details.

### Heart rate data

Heart rate values were collected at 1 Hz frequencies and narrowed to averages of non-overlapping 5-s intervals ^11^. All HR time series were aligned with their respective game time series and then trimmed to include only data between the beginning and the end of each game. Through summary statistics and visual inspections, each time series was examined to identify any data distortion. A square filter with a lower limit of 30 bpm and upper limit of 180 bpm and a Hampel filter with a window size of five intervals for detection of local outliers were applied in each sub-series.

We used Multidimensional Recurrence Quantification Analysis (MdRQA)^34^ to measure collective synchrony among participants’ heart rates at each game. MdRQA is a recurrence-based technique created to analyze the group-level behavior of assemblies larger than a dyad by quantifying patterns of repetition in a multidimensional time-series dataset. The value of this analysis relies on its potential to handle complexity by analyzing each timeseries not from its singular perspective but as an entity whose actions are part of a dynamically interacting system. Although this specific technique is relatively new, MdRQA belongs to a family of techniques known as Recurrence Quantification Analysis, which are designed to measure the coordination patterns of multiple variables across time^34^. While there are other methods for analyzing multiple dimensions within a system (e.g., neural networks), recurrence analyses are particularly suitable for understanding systems that are not already well-known to the researcher. These techniques have been recently applied to the study of dynamical physiological systems in the context of various behaviors and activities^45^.

MdRQA results in a recurrence plot generated for each game. Beyond the visual representation of synchrony, this plot allows the extraction of a number of recurrence measures that can be used to quantify group synchrony. Although these measures are inter-related, they each capture a different aspect of the dynamical relationship between the system’s variables. For the purposes of our study, we looked at recurrence rate (REC), determinism (DET), and average diagonal line length (ADL). REC refers to the sum of all recurrent points in the recurrence plot divided by the size of the recurrence plot. DET, an estimate of system predictability, measures the percentage of recurrent points that are diagonally adjacent. ADL, an estimate of prediction time, quantifies the average length of diagonal lines formed by recurrent points in the recurrence plot^34,36,37^.

Like other recurrence quantification analyses, MdRQA requires embedding time series into a phase space obtained by a time-delay method. We applied the Average Mutual Information (AMI) and False Nearest Neighbor (FNN) methods^46^ to obtain the delay and embedding parameters for each time series. Since the values of the delay and embedding parameters vary slightly among time series, we picked the first integer value right above the average for each parameter (delay = 9, embedding dimension = 4)^34^. Each time series was normalized to reduce the influence of the signal’s magnitude^47^.

In our study, the number of participants varied across games. This has an impact on the choice of the radius value, which increases in a square root relation to the embedding dimension, and therefore affects the interpretation of the MdRQA outcomes^35^. To overcome this problem, we analyzed up to 20 combinations between triads of participants (as the minimal number of participants in a single game was three) within each game. We applied a resampling MdRQA on each sub-group of each game by setting a fixed range of acceptable values for %REC (5.00–5.20%) across games. We then took the average values of all the resampling MdRQA outcomes per game. Comparing this to a standard MdRQA approach, we found that both DET (r = 0.807, *p* < 0.001) and ADL (*r* = 0.783, *p* < 0.001) were strongly correlated across the two methods. Re-running the analyses using the standard metrics produced no differences in the results (see Table S2).

Independent samples t-tests were conducted to compare the MdRQA outcomes between the two conditions (In-person vs. Remote). As the assumptions of normality and homoscedasticity of variances were not met, we opted for more conservative Welch tests. Analyses of Covariance (ANCOVA) were performed to examine the interaction between condition and synchrony.

### Game properties

To adjust for structural effects of the game, we created a coding scheme consisting of 14 key game actions. Codes were ascribed for successful field goals or free throws, failed field goal or free throw attempts, slam dunks, blocks, fouls, steals, rebounds, turnovers, time outs, and halftime breaks (see Table S3). A group of five research assistants watched each game and coded for the occurrence and timing of each event by filling a *Game Coding Matrix* with the codes of the events occurring at the particular game time and time of the day. For each game, the coding was checked by three different researchers. This allowed us to compute the total number of events per game and use it as a control variable in our robustness checks.

A comparison between games in the two conditions showed there were no differences between wins and losses, although there were differences in the average final game score (*t*(104.75) = 7.998, *p* < 0.001; *d* = 1.20) and the total number of actions per game (*t*(42.56) = 2.225, *p* = 0.030; *d* = 0.46). To account for these differences, we adjusted for them in our models.

### Moderated mediation models

The moderated mediation models were run with 5000 boostraps (*N* = 181). All continuous variables contributing to the product (i.e., ADL and DET) were mean-centred. There were no main effects of condition on transformativeness (*p*’s > 0.260). We re-ran the moderated mediation model multiple times with relevant covariates to avoid overfitting the model with a relatively small sample size (Table S4). Replacing DET with ADL in these models largely produced results with similar *p* values and the Index of Moderated Mediation was consistently positive, i.e. the 95% CIs did not cross zero. The only exception was when including frequency of attendance as a covariate, where the effects were in the expected direction but the *a* path was only marginally significant (*p* = 0.050), Index of Moderated Mediation = 0.957, CI[− 0.016, 2.368].

## Supplementary Information

Supplementary Information 1.

Supplementary Information 2.

## Data Availability

The data analyzed for this study are available from the corresponding author on reasonable request.
