# Supplementary material for: Being in a crowd bonds people via physiological synchrony
Source: Sci Rep. 2022 Jan 12;12:613. doi: 10.1038/s41598-021-04548-2 (PMC8755740; doi:10.1038/s41598-021-04548-2)
Supplement: Supplementary file 1 — Supplementary Information 1. [file 41598_2021_4548_MOESM1_ESM.pdf]

Supplementary Materials for

Being in a crowd bonds people via physiological synchrony

G. Baranowski-Pinto[1]*, V. L. S. Profeta[2], M. Newson[3 4], H. Whitehouse[3], D. Xygalatas[5]*

Correspondence to: gabrielabaranowski@gmail.com; xygalatas@uconn.edu

Survey instruments

Basketball IQ index [1]

1. "The Paint" refers to what area of the court?

   A. The painted logo at the center of the court where the game begins with a jump ball.

   B. The backboard of the basket (which used to be painted in the home team colors).

   C. The rectangular area under the basket extending to the free throw line.

   D. The area inside the 3-point arc.

2. How many points are awarded when a shot is made close to the basket?

   A. 1 point

   B. 2 points

   C. 3 points

   D. 4 points

3. "Free Throws" are

   A. awarded for any kind of foul (offensive or defensive) involving illegal physical contact.

   B. shot from the exact location where the foul happened.

   C. scored with an escalating point value as the game goes on.

   D. awarded automatically if an offensive player is in the act of shooting when fouled.

4. A “shot-clock violation” occurs when

A. the team who possesses the ball fails to attempt a field goal before the shot clock expires.

B. a player stands in an area under the basket longer than 3 sec without shooting.

C. a player holds the ball for more than 5 sec when being closely guarded.

D. a team fails to take a shot before the 10 sec shot clock expires after crossing mid-court.

Sport Spectator Identification Scale (SSIS) [2]

Please select the point on the scale that best represents your answer.

1. How much time do you spend per week with matters related to the UConn Basketball team (e.g., following news, discussing related matters, etc.)? (1= No time, 7= A lot of time)
2. How closely do you follow UConn Basketball team in person? (1= Never, 7= Almost every day)
3. How closely do you follow UConn Basketball team on the printed and online media (e.g., TV, radio, newspaper, internet, etc.)? (1= Never, 7= Almost every day)

Identity Fusion Scale [3]

Please select the point on the scale that best represents you. (1= Strongly disagree, 7= Strongly agree)

1. The UConn Basketball team is me
2. I am one with the UConn Basketball team
3. I feel immersed in the UConn Basketball team
4. I have a deep emotional bond with the UConn Basketball team
5. I am strong because of the UConn Basketball team
6. I’ll do for the UConn Basketball team more than any other UConn Basketball fan would

7. I make the UConn Basketball team strong

Frequency of Game attendance

1. How many UConn Women's Basketball games have you attended last season (2015-2016)?

2. How many UConn Men's Basketball games have you attended last season (2015-2016)?

3. How many UConn Women's Basketball games do you think you will attend this 2016-2017 season?

4. None How many UConn Men's Basketball games do you think you will attend this 2016-2017 season?

Transformativeness scale [4]

To what extent has today's game shaped you as a person?

a) 1= The person I am now is absolutely unrelated to this experience, 7= I am who I am today because of this experience.

b) 1= This experience has played no role in shaping the person I am today, 7= This experience has played a defining role in shaping the person I am today.

c) 1= If I had not had this experience, I would still be the exact same person, 7= If I had not had this experience, I would be an entirely different person.

Pictorial Identity Fusion Scale [5]

Select the point on the scale that best represents you right now.

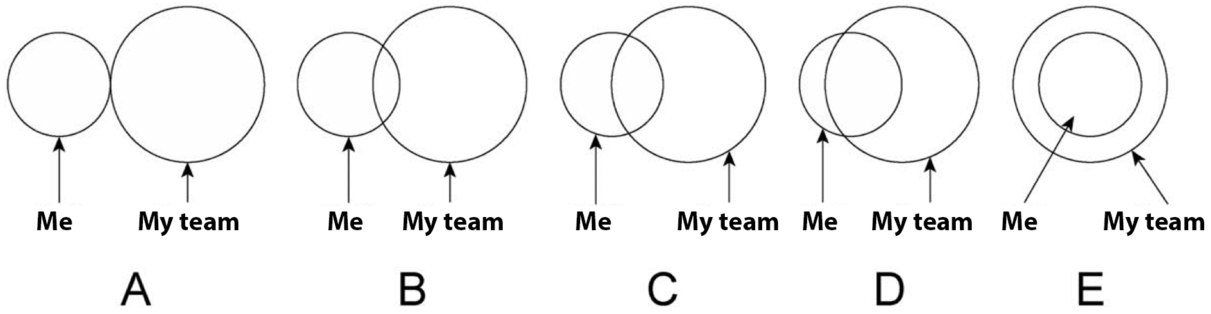
Me
My team
A
Me
My team
B
Me
My team
C
Me
My team
D
Me
My team
E

	In-person	On-screen	Total
**N**	147	35	182
**Age**	22.61 (10.64)	19.40 (1.96)	21.99 (9.67)
**Gender**			
Female	70	16	86
Male	77	19	96
**Ethnicity**			
Asian	14	10	24
Black	8	2	10
Latino/Hispanic	4	0	4
Native Hawaiian/Pacific	1	0	1
White	111	21	132
Bi-racial	9	2	11
**Education**			
Some College	115	30	145
College Degree	21	4	25
Graduate Degree	10	1	11
**Baseline Fusion (z-scored)**	.00 (1.03)	-.03 (.88)	.00 (1.00)
**Basketball IQ (0-4)**	3.46 (.82)	3.63 (.60)	3.49 (.79)
**SSIS (3-21)**	13.50 (3.83)	12.91 (3.59)	13.39 (3.78)
**Attendance**	3.73 (1.81)	3.66 (2.04)	3.71 (1.85)

Table S1.
Demographic characteristics of our sample.

	Resampling MdRQA		Standard MdRQA	
	**In-person**	**On-screen**	**In-person**	**On-screen**
**REC**	5.150 (.020)	5.134 (.044)	5.072 (.061)	5.098 (.083)
**DET**	51.659 (4.992)[a]	45.733 (4.483)	69.417 (9.045)[c]	47.694 (5.736)
**ADL**	2.733 (.135)[b]	2.565 (.082)	3.687 (.633)[c]	2.622 (.108)

Table S2.
Comparison of Resampling and Standard MdRQA procedures. Correlation between resampling and standard MdRQA of DET ($r = .807$, $p < .001$) and ADL ($r = .783$, $p < .001$). P-values for comparisons between In-person and On-screen within MdRQA procedures.*a* = $p < .05$, *b* = $p < .01$, *c* = $p < .001$.

	Action	Description
1	**2pt field goal**	A basket other than a free throw scored on any shot inside the three-point area.
2	**3pt field goal**	A basket scored outside of the three-point area.
3	**1pt field goal**	A free throw
4	**2pt attempt**	A failed attempt at a 2pt field goal
5	**3pt attempt**	A failed attempt at a 3pt field goal
6	**1pt attempt**	A failed attempt at a free throw (1pt goal)
7	**Dunk**	A slam dunk is a shot made by jumping in the air, controlling the ball above the horizontal plan of the rim, and scoring by putting the ball through the basket with one or both hands.
8	**Block**	When a defensive playing legally deflects a field goal attempt from an offensive player (without a foul).
9	**Foul**	An infraction of the rules consisting in illegal contact with an opponent and/or unsportsmanlike behavior.
10	**Steal**	When defensive player legally (without a foul) causes a turnover by his positive, aggressive action (e.g., by deflecting, controlling, or by catching an opponent's pass or dribble of an offensive player.
11	**Rebound**	When a player retrieves the ball after a missed field or free throw.
12	**Turnover**	When a team loses possession of the ball to the opposing team before a player takes a shot at his/her team's basket.
13	**Time out/Half time**	Timeout: when a team chooses to stop the clock while in possession of the ball.<br>Half time: A game is divided into 4 quarters consisting of 10 minutes each. The halftime break period is fifteen minutes long.

Table S3.
Coding scheme for game actions.

Covariate	Index of Moderated Mediation	Significant changes to model
Baseline fusion	.017 CI[.002, .037]	Baseline fusion predicted transformativeness (.758, p = .015) and post-game fusion (.491, p < .001).
Demographics		
**Age**	.027 CI[.003, .055]	Age predicted transformativeness (-.066, p = .049).
**Gender**	.027 CI[.004, .057]	N.s. throughout.
**Attendance**	.021 CI[.001, .047]	Attendance predicted transformativeness (.404, p = .017) and post-game fusion (.114, p = .004).
Game properties		
**Team gender**	.026, CI[.004, .054]	N.s. throughout.
**Number of events**	.027, CI[.004, .054]	N.s. throughout.
**Score**	.027 CI[.004, .053]	N.s. throughout.

Table S4.
Moderated Mediation results. Including demographic variables and game properties as covariates did not reduce the strength of the model.

1. Blau, J. J. C., Petrusz, S. C. & Carello, C. Fractal Structure of Event Segmentation: Lessons From Reel and Real Events. *Ecol Psychol* **25**, 81–101 (2013).

2. Wann, D. & Branscombe, N. Emotional responses to the sports page. *Journal of Sport and Social Issues* **16**, (1992).

3. Gómez, Á. *et al.* On the Nature of Identity Fusion: Insights Into the Construct and a New Measure. *J Pers Soc Psychol* **100**, 918–933 (2011).

4. Newson, M., Buhrmester, M. & Whitehouse, H. Explaining Lifelong Loyalty: The Role of Identity Fusion and Self-Shaping Group Events. *Plos One* **11**, e0160427 (2016).

5. Swann, W. B., Gómez, A., Seyle, D. C., Morales, J. F. & Huici, C. Identity fusion: the interplay of personal and social identities in extreme group behavior. *J Pers Soc Psychol* **96**, 995–1011 (2009).
